# Transcriptome analysis of *Phoenix canariensis* Chabaud in response to *Rhynchophorus ferrugineus* Olivier attacks

**DOI:** 10.3389/fpls.2015.00817

**Published:** 2015-10-14

**Authors:** Antonio Giovino, Edoardo Bertolini, Veronica Fileccia, Mohamad Al Hassan, Massimo Labra, Federico Martinelli

**Affiliations:** ^1^Unità di Ricerca per il Recupero e la Valorizzazione delle Specie Floricole Mediterranee, Consiglio per la Ricerca in Agricoltura e l'Analisi dell'Economia AgrariaPalermo, Italy; ^2^Istituto di Scienze della Vita, Scuola Superiore Sant'AnnaPisa, Italy; ^3^Dipartimento di Scienze Agrarie e Forestali, Università degli Studi di PalermoPalermo, Italy; ^4^Istituto Euromediterraneo di Scienza e TecnologiaPalermo, Italy; ^5^Universitat Politècnica de València, Instituto de Biología Molecular y Celular de Plantas, Consejo Superior de Investigaciones Científicas (CSIC), Ciudad Politécnica de la Investigación (CPI)Valencia, Spain; ^6^Dipartimento di Biotecnologie e Bioscienze, Università degli Studi di Milano BicoccaMilano, Italy

**Keywords:** genes, palm, *Phoenix canariensis*, Red Palm Weevil, *Rhynchophorus ferrugineus*, RNA-seq

## Abstract

Red Palm Weevil (RPW, *Rhynchophorus ferrugineus* Olivier) threatens most palm species worldwide. Until now, no studies have analyzed the gene regulatory networks of *Phoenix canariensis* (Chabaud) in response to RPW attacks. The aim of this study was to fill this knowledge gap. Providing this basic knowledge is very important to improve its management.

**Results:** A deep transcriptome analysis was performed on fully expanded leaves of healthy non-infested trees and attacked trees at two symptom stages (middle and late infestation). A total of 54 genes were significantly regulated during middle stage. Pathway enrichment analysis showed that phenylpropanoid-related pathways were induced at this stage. More than 3300 genes were affected during late stage of attacks. Higher transcript abundances were observed for lipid fatty acid metabolism (fatty acid and glycerolipids), tryptophan metabolism, phenylpropanoid metabolism. Key RPW-modulated genes involved in innate response mediated by hormone crosstalk were observed belonging to auxin, jasmonate and salicylic acid (SA) pathways. Among transcription factors, some WRKYs were clearly induced. qRT-PCR validation confirmed the upregulation of key genes chosen as validation of transcriptomic analysis.

**Conclusion:** A subset of these genes may be further analyzed in future studies to confirm their specificity to be induced by RPW infestations.

## Introduction

*Rhynchophorus ferrugineus* Olivier (commonly known as the Red Palm Weevil, RPW) is considered the worst pest threat for palm species worldwide. More than 30 palm species are attacked by this insect, including *Phoenix canariensis* Chabaud, *P. dactylifera* L., and *Cocos nucifera* L. (Ju et al., 2011). RPW was first reported in tropical Asia and then spread worldwide, reaching several Middle Eastern countries, Africa and the Mediterranean basin in the 1980s (Faleiro, 2006). In Europe, including Spain, especially the Canary Islands, and Southern Italy, RPW inflicted great economic damage in areas where palm distinguishes the scenic beauty of the public and private gardens of the cities and countryside. In the United States a species of RPW has been identified (Rugman-Jones et al., 2013). RPW belongs to the Curculionidae family of Coleoptera. The damage caused to palm is due to the large larvae forming large tunnels within soft and terminal plant tissues. The larvae can achieve a size of 5 cm, and different generations can be present in the same infected tree. Adults may deposit approximately 200 eggs at the base of leaves or in wounded tissues, and larvae migrate within all parts of trees, including the roots, destroying all the plant organs, and structures (Gutierrez et al., 2010). A major issue in the management of pest attacks is the late detection of infestations. Usually, symptoms are only visible when the larvae have reached the latest developmental stage and when plant organs are already compromised (Ju et al., 2011). Indeed, at this point, it is too late to save the infected trees. The symptoms of infestation include a gnawing sound caused by larvae feeding inside infested trees, chewed plant material at the external entrances of tunnels generating particular smells, the presence of empty pupal and dead adult bodies close to infested palms, the breaking of the palm crown and trunk (Faleiro, 2006) and the asymmetry of the palm crown. These symptoms are not visible prior to 5–6 months of infestation, when any prophylaxis is useless (Faleiro, 2006). The evaluation of the signs of larval mines and chewed material at the leaf bases is the typical way to detect signs of early infestation. However, early infestations can be difficult to detect in adult palms because actively growing portions are usually at the top of the plants. Therefore, a wide range of physical, chemical and biological methodologies are under development to detect the insect inside the palms as soon as infestations occur. Computer-assisted tomography shows interesting results for the inspection of the granary weevil in wheat (Haff and Slaughter, 2004). However, these methods are expensive and difficult to be applied in infected palms where the whole tree needs to be inspected. Indeed, these methods may not be used for the large-scale scouting of infestations. The analysis of volatiles that are emitted by the fermentation processes of infested trees was also addressed by means of trained dogs that recognized the characteristic odors of plants that were attacked by RPW (Nakash et al., 2000). However, the low selectivity and reliability due to the presence of several volatiles that are unrelated to the RPW infection limit the use of such methods (Mielle and Marquis, 1999). Bioacoustic sensors could represent an alternative system for early detection (Gutierrez et al., 2010). However, this detection is still expensive and requires trained technical staff to discriminate the RPW noise from others. Moreover, these methods are not very effective at early feeding phases. Until now, no large-scale analyses of palm responses to RPW attacks have been conducted. Transcriptomics represent a powerful tool not only to elucidate the physiological effects of RPW attacks on infected palms but also to the identify biomarkers that are usable to improve detection when symptoms are still unclear. In addition, transcriptomics is essential to develop therapeutics based on gene biotechnology. Next-Generation Sequencing (NGS) has revolutionized transcriptomic studies because this technology can detect rare and unknown transcripts and splice variants that are not present in microarrays, offering a more detailed and profound analysis of the transcriptome. The power of this method is fully exploitable when extensive genomic information is available for the organism under investigation. In plants, RNA-seq has proven effective in studying the transcriptomic profile after pathogenic infections (Martinelli et al., 2012, 2013; Chen et al., 2014; Zhang et al., 2014) and in identifying possible targets for their early detection (Tremblay et al., 2013).

Recent studies have been published on the genomic analysis of *P. dactylifera* L. A genome assembly was performed for Khalas, an elite cultivar (Al-Dous et al., 2011). The assembled sequence was approximately 380 Mb spanning mostly gene-rich regions (90% of genes were covered) and including >25,000 gene models. Another genome sequence of date palm is available (Al-Mssallem et al., 2013).

The aim of this study was to gain insight into gene regulatory networks of responses to RPW attack in *Phoenix canariensis*. This research will allow us to (1) clarify the gene regulation mechanisms of leaf metabolism in response to RPW attacks at different stage of infestations and (2) identify possible host biomarkers that may confirm RPW typical symptomatology.

## Materials and methods

### Plant material and experimental design

This study was performed on *Phoenix canariensis* Chabaud. We selected 15–20-year-old trees with a diameter of 70–90 cm. Analyzed leaves were 4–5 m long with 80–100 segments on each side of the spine. The plants were field-grown in a former cultivation of approximately 2 ha, located in Trabia (Palermo, Italy). These plants were genetically different although they have a similar genetic background since they were derived from the same mother plant. No specific permissions were required for these locations and activities. The field studies did not involve endangered or protected species. These are the GPS coordinates of the area where plants were analyzed: 38°00′00″N 13°39′00″E; 38°00′00″N 13°39′00″E.

The plants were arranged on the sides of a private driveway. The trees were divided into three groups: Healthy unattacked trees (He), stage 1 (the middle stage of infection—S1), and stage 2 (plants with late symptoms—S2). The pathogen presence was visually confirmed. No plants were available at very early stage of infection. Although trees at S1 showed symptoms, analyzed leaves were green and did not show any symptoms such as the healthy ones.

**Figure 1:**
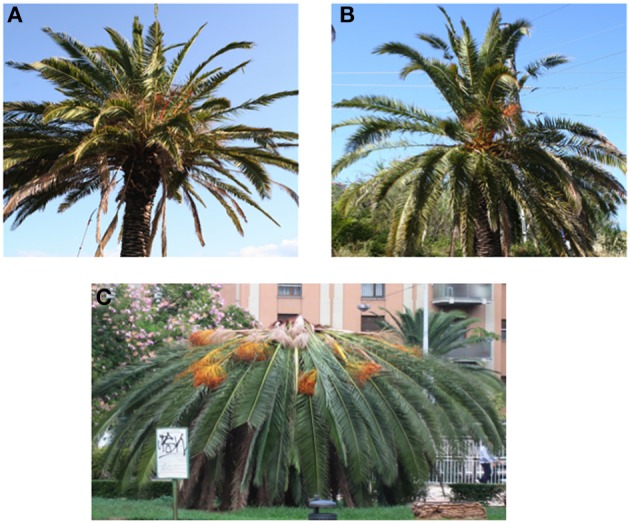
**Palm trees of the three analyzed categories: (A) healthy (He); (B) stage S1 (middle stage of infestation); (C) stage S2 (intense level of infestation)**.

The He category was composed of palm trees with no symptoms of Red Palm Weevil attacks and no signs of other common diseases or pathogen attacks (Figure 1). The sampled individuals showed fully expanded leaves without any sign of gnawing. To exclude sampling errors (i.e., sampling asymptomatic infested plants instead of healthy uninfected plants), we monitored the sampled He trees for the following 90 days from sampling. This period was defined based on the timing needed to show early symptoms of infestations (Ju et al., 2011). The S1 category represents trees that showed anomalous behavior of the canopy with the beginning of characteristic retracted asymmetry of the crown. Individuals showed fully expanded leaves without any sign of gnawing at the sampling time; however, after 30–40 days, some of the leaves showed a flattened and nibbled vegetative apex. Moreover, after 50 days, some larvae were detected in the S1 individuals. The S2 category included trees with loss of leaves for subsidence of foliar rachis. In these trees, most of the leaves showed the typical recline induced by RPW. Foliar desiccation was much more apparent than in S1. While analyzed leaves of S1 looked green as the healthy ones, S2 leaves showed evident yellowing.

Each of the three conditions was analyzed in duplicate; thus, a total of six samples (biological replicates) were analyzed by deep sequencing. Each biological replicate was composed of 10–15 leaf portions of three distinct trees. The leaf portions were collected from the middle of the palm leaves at the same time during the day. The samples were immediately frozen in liquid nitrogen and stored at −80°C.

### Analysis of the plant health status

The rhizosphere was analyzed to identify any other pathogenic organism in addition to RPW. The soil samples were collected from all four plant sides at a depth of 30 cm (500 g per plant). The pathogens were isolated through different traditional techniques. Substrates of the soil, roots and bark were prepared for both generic and selective pathogen isolation. The soil samples were repeatedly mixed, and 10 g was taken, dissolved in 100 ml of sterile distilled water and agitated for 15 min. A 1 ml aliquot of the suspension was inoculated in Petri dishes with cultured generic potato dextrose agar (PDA). The root and bark tissues were previously superficially sterilized and immersed in an aqueous solution of sodium hypochlorite at 10% for 3–5 min, rinsed in sterile distilled water and dried. The tissue fragments were placed on the culture substrates PDA, corn meal agar (CMA) plus streptomycin (20–30 mg/L) and PARPNH substrate-selective culture. All the plates were dark-incubated at 24 ± 1°C. The developed fungal colonies were grown in purity and characterized based on both macroscopic (morphology, color, and speed of growth of the colony) and microscopic features (fruiting bodies, spores, and conidia). The genera and species were identified based on dichotomous recognized keys. All the analyzed palms were tested for the presence of common bacterial and fungal pathogens (i.e., Penicillium sp., Alternaria sp., Mortierella sp., Aspergillus sp.). None of these common pathogens were present.

### RNA extraction

The total RNA from each biological replicate was isolated using a rapid RNA extraction method that was developed by Gambino et al. (2008). The RNA concentrations were determined using a NanoDrop ND-1000 spectrophotometer (NanoDrop Technologies, Wilmington, DE). The RNA quality and purity were assessed by an Agilent Bioanalyzer (Folsom, CA).

### RNA-seq analysis

The RNA samples were processed using the TruSeq RNA-seq sample prep kit from Illumina (Illumina, Inc., CA, USA). Briefly, the poly-A containing mRNA molecules were purified using poly-T oligo-attached magnetic beads and fragmented into small pieces using divalent cations at an elevated temperature. The cDNA was synthesized by reverse transcription, and standard blunt-ending plus add “A” was performed. Then, Illumina TruSeq adapters with indexes were ligated to the ends of the cDNA fragments. After the ligation reaction and separation of the unligated adapters, the samples were amplified by PCR to selectively enrich those cDNA fragments in the library with adapter molecules at both ends. The six samples (RNA pools of 10–15 leaves of three individual plants) were loaded into one lane of an Illumina flow cell, and clusters were created by Illumina cBot. The clusters were sequenced at ultra-high throughput on the Illumina HiSeq 2000 (Illumina Inc.). One lane in 6-plex was run, obtaining between approximately 21.1 and 29.8 million single reads per sample, each 50 bp long. The data were produced on an IGA Technology Services Srl (Udine, Italy) Illumina platform. All the sequenced reads were compared to the Date Palm Genome Draft Sequence Version 3.0 (http://qatar-weill.cornell.edu/). The gene prediction from the Date Palm Genome Draft Sequence Version 3.0 (file PDK30-mrna.fsa.gz downloaded from http://qatar-weill.cornell.edu/) was used. The Software CLC Genomic Workbench 5.5.1 (CLC Bio, Denmark) was used for reads trimming and alignment on the reference. The reads were quality trimmed using the modified-Mott trimming algorithm (parameter: trim using quality score = 0.05). The alignment on a reference parameters were set-up in CLC Genomics Workbench as following: Mismatch cost = 2; Insertion cost = 3; Deletion cost = 3; Length fraction = 0.9; Similarity fraction = 0.95. BLASTx was used to determine the date palm gene predictions of the putative orthologous *Arabidopsis thaliana* genes (*e* < 10^−4^). DESeq 2 package from Bioconductor in the R statistical software suite (Love et al., 2014) was used to estimate the euclidean sample distance and the expression level of transcripts among different conditions. DESeq 2 program performs normalization, variance estimation and differential expression of the raw read counts and works best with experiments with replicates. The log_2_-fold ratio and adjusted *p*-values (FDR) based on the t-distribution for each gene for the two pairwise comparisons (He-S1 and He-S2) were calculated. Genes with a log_2_-fold ratio >1 or < −1 and with an adjusted *p*-value (FDR) below 0.1 were considered differentially expressed. RNA-seq and details of the samples were submitted on Squence Read Archive (NCBI) (SAMN04031658).

### Functional categorization of the predicted RPW-regulated transcripts

The sequenced transcripts were mapped to the Date Palm Genome Draft Sequence Version 3.0 and used as a reference genome. For each of the palm genes, the gene length, unique and total gene reads, annotation and RPKM expression values were obtained. The closest *Arabidopsis* putative orthologs were determined for each gene to allow the use of functional genomics tools. The integrated data-mining approach used different web tools, such as MapMan, PageMan, PathExpress, and Cytoscape to dissect the transcriptome responses and decipher the gene regulatory networks. A list of predicted transcripts that were differentially expressed at a significant level (*p* < 0.05 and an absolute value of log_2_-fold change >1 or < -1) was obtained for each of the two pairwise comparisons (S1 vs. He and S2 vs. He). These input data files were used for all the data mining tools.

The functions of the differentially expressed genes (as *Arabidopsis* putative orthologs) were visualized using the MapMan web-tool (Thimm et al., 2004; http://mapman.gabipd.org/web/guest/mapman) through the Ath_AGI_isoform_model_Tair10_Aug2012.m02 mapping file that was downloaded from the MapMan server. The PageMan visualization tool was used for GSEA analysis using the Wilcoxon test (no correction and ORA cutoff = 1.0). A pathway enrichment analysis was performed using PathExpress (Goffard and Weiller, 2007) using the *Arabidopsis* putative orthologs of the differentially expressed palm transcripts.

### RT-PCR validation

Nine genes were chosen for qRT-PCR validation of RNA-seq data. Four biological replicates were considered for each of three conditions (Healthy, S1, and S2). Each replicate was a pool of 10–15 mature leaves from the same plant. The four chosen plants belonged to those used for RNA-seq analysis: two belonged to the first replicate and the other two belonged to the second replicate of deep sequencing analysis. Primers were designed basing on each target sequence using Primer Express software (Applied Biosystems, Foster City, CA, Table S1). RNA was extracted as previously described. Retrotranscription was performed following the Quantitect Reverse Transcription Kit (Qiagen) instructions. A standard curve was generated for each gene. Amplifications used 25 ng cDNA in a 15 μL final volume were performed on a Biorad iQ5 PCR system (Biorad) using standard amplification conditions: 10 min at 95°C; 40 cycles of 15 s at 95°C; and 1 min at 60°C. All PCR reactions were performed in duplicate (technical replicates). Fluorescent signals were collected during the annealing step and C_T_ values extracted with an auto calculated threshold followed by baseline subtraction. *18S* (AF206991.1) was used as an endogenous reference and ΔΔ*C*_T_ was calculated by subtracting the average of *18S* from the average *C*_T_ of the gene of interest. The reference gene was tested on the 12 analyzed samples and any significant changes of expression were observed between the three samples categories.

## Results

### Illumina RNA sequencing

RNA-seq was used to evaluate palm responses to RPW attack by comparing the expression profiles of healthy trees (He) with infected trees during the middle and late stages of infection (S1 and S2). The genome sequence of *Phoenix dactylifera* was used as reference genome for sequence annotation (Al-Dous et al., 2011). This allowed obtain a list of palm genes correspondent to the assembled sequences. Then we identified the correspondent *Arabidopsis* ortholog to each of these genes. We obtained a total of 21.3–29.8 million raw reads for the six palm samples (Table 1). Of them 21.1–29.5 were trimmed. A total of approximately 8.6–12.3 million 50-nt single-end reads from each cDNA library were uniquely mapped to the genome published by Al-Dous et al. (2011). Unique mapped reads were approximately 40.6–41.6 % of the corresponding total trimmed reads per sample. Multiple mapped reads were approximately 1.1 % and they were discarded from the analysis.

**Table 1:** **Number of raw reads, trimmed raw reads, mapped reads (unique), mapped reads (multiple)**.

**Sample**	**Healthy 1**	**Healthy 2**	**Stage 1-1**	**Stage 1-2**	**Stage 2-1**	**Stage 2-2**
Raw reads	21,333,300	25,657,081	22,350,317	29,707,097	29,781,177	26,491,474
Trimmed raw reads	21,139,613	25,438,175	22,142,923	29,456,600	29,527,217	26,123,111
Mapped reads (unique)	8,591,083	10,460,021	9,023,479	12,174,309	12,291,541	10,860,082
% of mapped reads (unique)	40.64	41.12	40.75	41.33	41.63	41.57
Mapped reads (multiple)	227,933	268,690	233,928	337,254	337,657	297,427
% of mapped reads (multiple)	1.08	1.06	1.06	1.14	1.14	1.14

*Percentages of mapped reads (unique or multiple) were calculated from trimmed raw reads*.

**Figure 2:**
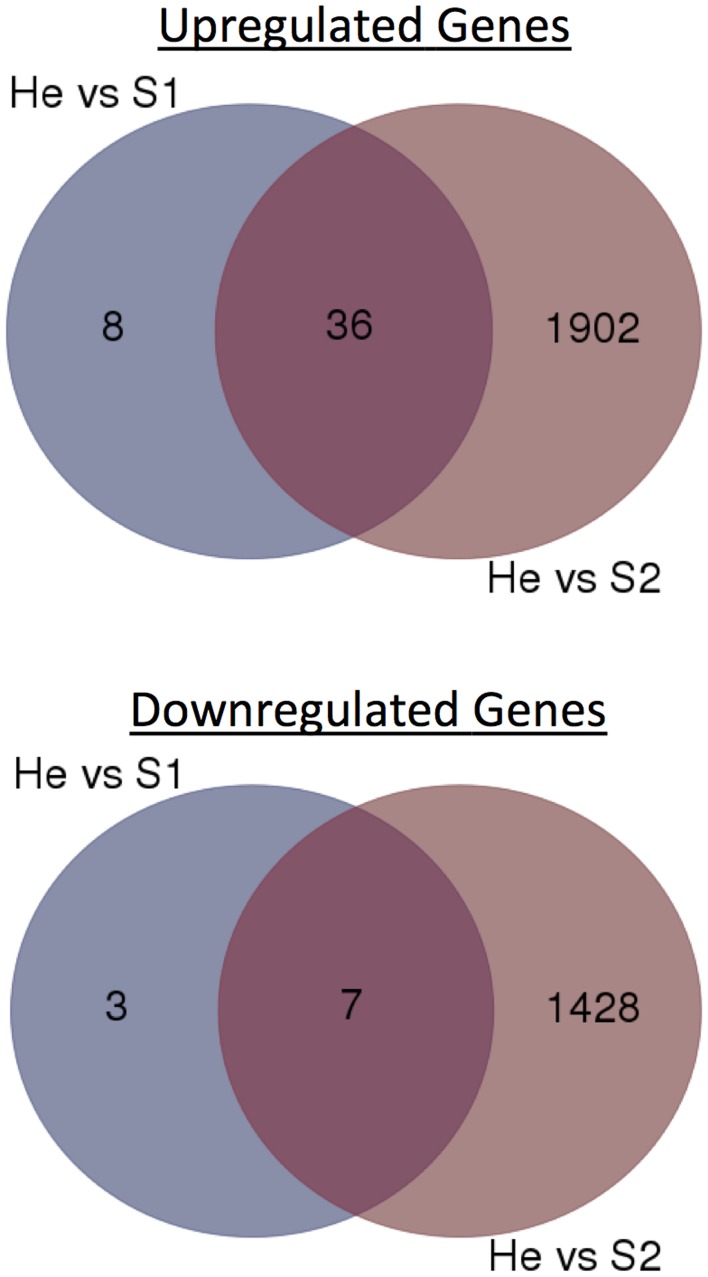
**Venn diagrams of RPW-regulated genes at He vs. S1 and He vs. S2 comparisons**. Numbers of up- or down-regulated genes were shown.

**Figure 3:**
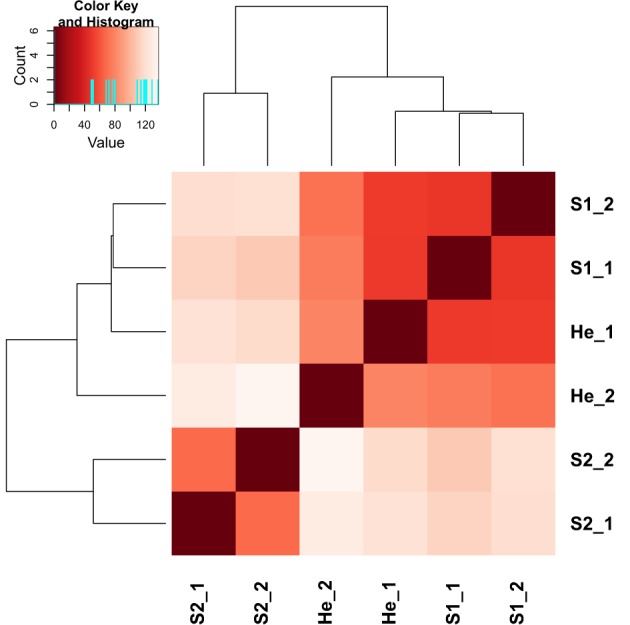
**A dendrogram based on mapped read counts was constructed for the six analyzed samples belonging to Healthy (He), Stage 1 (S1)—middle stage of infestation, Stage 2 (S2)—intense level of infestation**.

**Figure 4:**
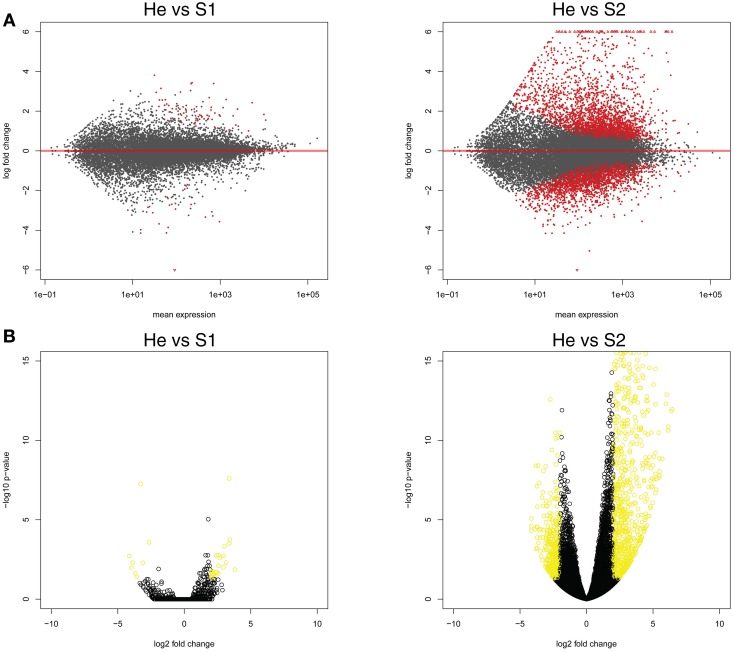
**(A)** Expression changes for each analyzed palm gene in the two pairwise comparisons (He vs. S1 and He vs. S2). In abscissa axis mean expression and in ordinate axis log fold changes were shown. **(B)** Volcano plots for both He/S1 and He/S2 comparisons were shown. In abscissa axis log_2_ fold change while in ordinate axis log_10_ *p*-value were shown for each identified gene.

The gene expression data of the two pairwise comparisons (S1 vs. He and S2 vs. He) were provided in Tables S2, S3. Figure 2 showed a Venn diagram of up- and down-regulated genes by RPW in both pairwise comparisons. A total of 44 genes were upregulated, and 10 genes were downregulated in S1 vs. He comparison. Most of them were reported in Table 2. A total of 3373 genes were differentially expressed in the S2 vs. He comparison (approximately 13.5 % of the predicted genes in the genome reference); 1938 were upregulated, and 1435 were downregulated. A dendrogram was constructed based on the overall transcriptome analysis of the six analyzed samples (Figure 3). As expected He and S1 were grouped together while S2 showed to be clearly distinguished by the other two sample categories. Volcano plots were performed for both S1/He and S2/He comparisons (Figure 4). In the S1/He comparison, although many genes have a log fold ratio higher than 1, they had a non-significant FDR. In the S2/He comparison a considerable portions of the analyzed genes were significantly regulated. These genes were mainly those that presented a higher fold ratio.

**Table 2:** **List of the main differentially regulated genes during stage 1 (FDR < 0.1)**.

**Annotation**	**AGI**	**Log_2_ fold ratio**
**REDOX PATHWAYS**		
Peroxidase superfamily protein	AT1G05260.1	−4.0
Peroxidase superfamily protein	AT1G68850.1	2.4
(2OG) and Fe(II)-dependent oxygenase protein	AT5G05600.1	3.0
(2OG) and Fe(II)-dependent oxygenase protein	AT4G10490.1	1.7
**LARGE ENZYME FAMILIES**		
GDSL-like Lipase/Acylhydrolase protein	AT1G74460.1	2.6
Glutathione S-transferase TAU 18	AT1G10360.1	2.0
Cytochrome P450 superfamily protein	AT5G07990.1	−3.9
Cytochrome P450 family 94 C polypeptide 1	AT2G27690.1	3.4
**SIGNAL PERCEPTION AND SIGNALING**		
Protein kinase superfamily protein	AT1G18670.1	1.6
Calcium-dependent protein kinase 16	AT2G17890.1	1.5
Protein of unknown function (DUF604)	AT2G37730.1	−3.3
Protein of unknown function (DUF616)	AT1G53040.1	1.6
Calcium-dependent protein kinase 28	AT5G66210.2	2.0
**TRANSCRIPTION FACTORS**		
WRKY40	AT1G80840.1	2.8
WRKY51	AT5G64810.1	2.7
NAC domain containing protein 32	AT1G77450.1	1.8
**PROTEIN MODIFICATIONS**		
RING/U-box superfamily protein	AT1G78420.1	−7.6
RING/U-box superfamily protein	AT1G53820.1	3.1
RING/U-box superfamily protein	AT2G18650.1	−4.1
U-box domain-containing protein kinase protein	AT2G45910.1	1.6
**SECONDARY METABOLISM**		
Cinnamate-4-hydroxylase	AT2G30490.1	3.0
Laccase 7	AT3G09220.1	2.9
Laccase 12	AT5G05390.1	2.3
Chalcone and stilbene synthase protein	AT5G13930.1	1.8
Leucoanthocyanidin dioxygenase	AT4G22880.1	−3.6
**DEFENSE RESPONSES**		
Disease resistance family protein / LRR protein	AT2G34930.1	1.7
Pathogenesis-related thaumatin protein	AT2G17860.1	1.8
Lipid-transfer protein/seed storage 2S albumin	AT3G22600.1	2.5
**CELL WALL MODIFICATIONS**		
Xyloglucan endotransglycosylase 6	AT4G25810.1	2.1
Beta-1 3-glucanase 5	AT5G20340.1	2.1
D-arabinono-1 4-lactone oxidase family protein	AT2G46740.1	3.4
Pectin lyase-like superfamily protein	AT3G62110.1	−3.7
**HORMONE-RELATED**		
Ethylene response factor 110	AT5G50080.1	1.2
Auxin amidohydrolase	AT1G51760.1	3.4
**OTHERS**		
Nitrate transporter 1.5	AT1G32450.1	1.3
LOB domain-containing protein 1	AT1G07900.1	2.2
Amino acid permease 3	AT1G77380.1	1.8
Formin homology5	AT5G54650.1	1.5
HXXXD-type acyl-transferase protein	AT5G41040.1	2.4
Alpha/beta-Hydrolases superfamily protein	AT2G39420.1	3.4
P-loop nucleoside triphosphate hydrolases protein	AT3G45080.1	−2.7
Glutamate receptor 2.8	AT2G29110.1	1.5
Glutamate receptor 2.7	AT2G29120.1	1.8
Di-glucose binding protein Kinesin motor domain	AT2G22610.1	−3.1
Integrase-type DNA-binding protein	AT4G36920.1	−1.9
Glycosyl hydrolase superfamily protein	AT4G16260.1	2.1

*The Log FD (Fold Ratio) was indicated. The complete list with the palm gene ID and Arabidopsis putative orthologsis available in the supplemental material (Table S2)*.

### Metabolic pathway and gene set enrichment analysis

The metabolic pathway enrichment analysis indicated that at stage S1 of infestation, phenylalanine metabolism, phenylpropanoid biosynthesis, flavonoid biosynthesis, and xenobiotic metabolism mediated by cytochrome P450 were significantly upregulated by RPW attacks (Table 3). In contrast, flavone and flavonol biosynthesis were significantly repressed at S1.

**Table 3:** **PathExpress analysis of the up and downregulated genes in each of the two pairwise comparisons**.

**S1 vs. He**	***P*-values**
**UPREGULATED**	
Phenylalaninemetabolism	4.1 × 10^−3^
Phenylpropanoidbiosynthesis	5.1 × 10^−3^
Flavonoidbiosynthesis	7.9 × 10^−3^
Metabolism of xenobiotics by cytochrome P450	2.6 × 10^−2^
**DOWNREGULATED**	
Flavone and flavonolbiosynthesis	1.3 × 10^−2^
**S2 vs. He**	***P*****-values**
**UPREGULATED**	
Fatty acid metabolism	3.3 × 10^−4^
Sphingolipid metabolism	3.6 × 10^−2^
Glycerolipid metabolism	3.7 × 10^−2^
Tryptophan metabolism	0.05
Alkaloid biosynthesis	0.05
**DOWNREGULATED**	
Starch and sucrosemetabolism	2.1 × 10^−3^
Phosphatidylinositol signaling system	3.6 × 10^−3^
Inositol phosphate metabolism	9.3 × 10^−3^
Glycosaminoglycan degradation	0.04

*Pathways with a P < 0.05 (no corrections) were affected by the RPW attacks. He, Healthy; S1, stage 1; S2, stage 2*.

At S2, RPW significantly induced lipid-related pathways such as fatty acid, sphingolipid, glycerolipid metabolism. In contrast RPW repressed starch and sucrose metabolism and inositol-related pathways.

**Figure 5:**
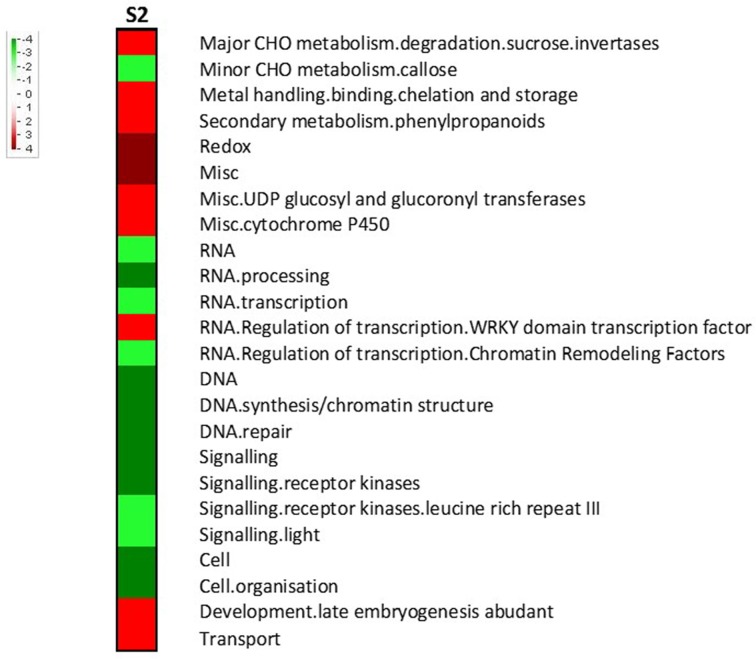
**Gene set enrichment analysis of the transcriptomic changes during stage 2 (S2)**. Pageman web-tool was used. Wilcoxon test with ORA cut off = 1 was used. A scale bar between -4 and 4 was chosen. Increased intensity of red and green respectively represented higher level of upregulation and downregulation.

Key primary metabolism pathways were influenced by RPW at S2, such as the sucrose (upregulated) and callose metabolism (downregulated) (Figure 5). Interestingly key players in transcription regulation of biotic stress responses were upregulated such as WRKYs. Genes encoding receptor kinases (including leucine rich repeat III) and cell organization-related proteins were repressed.

### Secondary metabolism, transcription factors, signaling, redox

At S1, some genes involved in phenylpropanoids were upregulated (Table 2). At S2, a high number of genes belonging to secondary metabolism were induced. Some phenylpropanoid genes were strongly activated during the late stage S2 such as O-*methyltransferasefamily protein 1* and *2, 3-coumarate-CoA ligase, ferulic acid 5-hydroxylase 1, cinnamoyl-CoA reductase*.

A list of differentially regulated genes at S2 encoding transcription factors was provided (Table S4). Regarding signal perception and transduction signaling, different classes of receptor kinases showed different trends of expression (Table S4). At S1, genes encoding a specific category of signal receptors DUF were differentially regulated: *DUF616* was slightly enhanced and *DUF604* was repressed (−3.3). At S2, most of the genes of DUF26 protein kinases and LRK10-like (serine/threonine protein kinases) were more abundant while leucine-rich repeat III, VI, and XII were mostly repressed. The transcript abundance of genes of large enzyme families (thioredoxin-related and cytochrome-related) that are involved in redox was severely affected by RPW attack.

### Hormone-related pathways

At S1, *Auxin amidohydrolase* and *ethylene response factor 110* were slightly higher in abundance. At S2, significant transcriptional changes were observed for a group of genes that are involved in auxin signal transduction (*PIN2* and *PIN4*) (Table S4). ABA-related pathways were affected as demonstrated by the induction of *HVA22J* and two *GRAM-domain-containing proteins*. In contrast, the expressions of *ABA1* and *AREB3* were lower at S2 compared to He. In general hormone-related pathways were drastically affected by RPW at S2. Two *ACC oxidases* that are involved in ethylene biosynthesis were upregulated. Two cytokinin receptors were significantly repressed, *HK2* and *HK3*. Some significant increases in transcript abundance were observed for genes that were involved in jasmonate synthesis (*allene oxide synthase* and *OPR2*) and salicylic acid-mediated response (glucosyltransferases).

### Biotic stress responses

**Figure 6:**
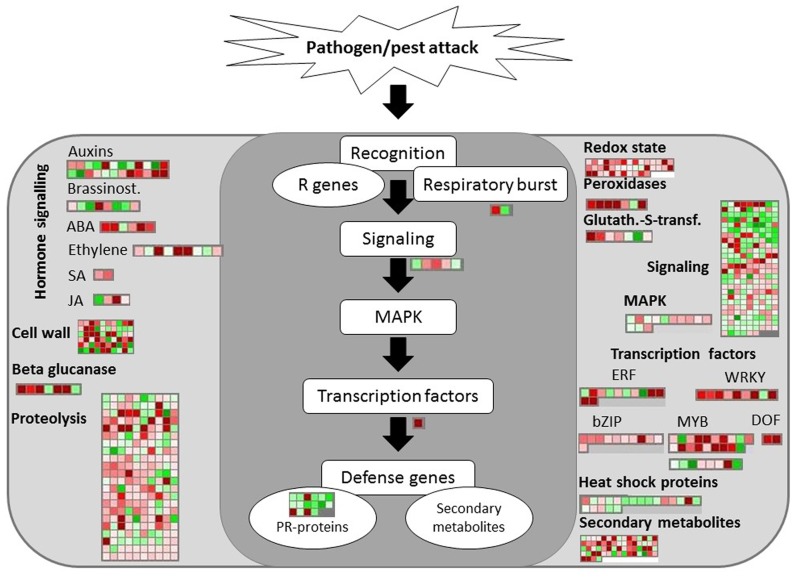
**Transcriptomic changes involved in biotic stress responses at stage S2**. The same scale bar used for the other figures was shown. Negative values represented repressed genes (green) at S2 in comparison to He and positive values (red) represented upregulated genes.

Defense-related pathways were activated at S1 as shown by the upregulation of genes encoding a *pathogenesis-related thaumatin protein* and a *disease resistance/LRR protein*. An overview of the transcriptomic changes involved in biotic stress pathways at S2 was provided (Figure 6). Extensive induction of genes encoding enzymes involved in redox state and peroxidases was observed in response to RPW at both disease stages. Two peroxidase genes were differentially regulated at S1: one was induced (Log FD = 2.4) and the other was repressed (Log FD = −4.0). Key genes encoding antioxidant enzymes were slightly upregulated at S1 (Table 2).

As far as it concerns, cell wall modification genes were upregulated at S1. Two laccase genes were induced at both S1 and S2. At S2, five genes involved in cell wall restructuring and encoding five *beta-1,3,- glucan hydrolases* were enhanced.

Pathogenesis-related (PR) proteins were mostly repressed although three disease-resistance family proteins were upregulated at late symptomatic stage. At S1, two WRKYs were upregulated. At S2, several *WRKY* genes were clearly enhanced, such as *WRKY9, WRKY14, WRKY40, WRKY47, WRKY28, WRKY72, WRKY75*, and *WRKY51*. In contrast, *WRKY2* was downregulated.

### qRT-PCR analysis

qRT-PCR were performed to validate expression trend of nine RPW-regulated genes (Table 4). Statistical differences of 15 of the 18 pairwise comparisons of gene expressions observed by RNA-seq were confirmed by qRT-PCR analysis. *Alpha-amylase* and *invertase* belonged to sugar and starch metabolism. RNA-seq analysis showed that both these two genes were not regulated at stage S1 while they were induced at stage S2. qRT-PCR confirmed data on S2 but showed that they were also significantly induced at S1. *UDP-glycosyltransferase* was confirmed to be induced at S2 while *UDP-glucose-SA glycosyltransferase* was enhanced at both S1 and S2. *Laccase7*, involved in secondary metabolism, was upregulated at S1. *Auxin amidohydrolase* was upregulated at both stages of infestation. *WRKY72* and *WRKY75* were confirmed to be higher in abundance at S2.

**Table 4:** **qRT-PCR analysis of 9 chosen genes**.

**Date palm ID**	**Description**	**ANOVA (RNA-seq)**		**ANOVA (qRT-PCR)**	
		**He vs. S1**	**He vs. S2**	**He vs. S1**	**He vs. S2**
PDK_30s654931g003	Alpha-Amylase	n.s.	6.4 (^*^)	1.5 (^*^)	4.9 (^*^)
PDK_30s705371g005	UDP-Glycosyltransf.	n.s.	5.6 (^*^)	n.s.	80.9 (^*^)
PDK_30s888011g002	Laccase 7	2.9 (^*^)	n.s.	0.37 (^*^)	n.s.
PDK_30s835131g001	WRKY 75	n.s.	7.5 (^*^)	n.s.	4.3 (^*^)
PDK_30s883821g030	UDP-glucose-SA glycosyltransf.	n.s.	1.5 (^*^)	2.4 (^*^)	3.2 (^*^)
PDK_30s936891g001	WRKY 40	2.8 (^*^)	2.7 (^*^)	9.2 (^*^)	1.4 (^*^)
PDK_30s1151561g006	Invertase	n.s.	3.0 (^*^)	0.8 (^*^)	1.4 (^*^)
PDK_30s1173851g004	Auxin amidohydrolase	3.4 (^*^)	1.7 (^*^)	10.1 (^*^)	1.2 (^*^)
PDK_30s1138471g004	WRKY 72	n.s.	3.3	n.s.	0.7 (^*^)

*Statistical analysis using ANOVA (P < 0.05) was shown*:

* *means significant, n.s. means not significant. Log_2_ fold ratio were indicated*.

## Discussion

In this work, we addressed the lack of knowledge on transcriptomic responses to RPW attacks. Plant secondary metabolites are well-known compounds involved in plant-insect interactions (Becerra, 2007). Phenylpropanoid genes were strongly affected by RPW attacks. This evidence highlighted that palms are activating counteracting responses to RPW from the middle stage of infection. We observed that BACT3 and IIL1 genes and other genes (*allinase* and *TAR2*) involved in the biosynthesis of sulfur-containing compounds were enhanced. These compounds have well-known defensive properties. Plants have been engineered to produce a cyanogenic glycoside, obtaining enhanced resistance to Phyllotretanemorum in *Arabidopsis* (Tattersall et al., 2001). These genes were not induced at S1 implying that palm responses to RPW attacks are activated too late and in an inefficient manner. Genes encoding strictosidine synthases were clearly enhanced only in response to RPW during the late stage. These genes are involved in alkaloid metabolism, a pathway that is generally stimulated in defense against insect herbivory. Flavonoids are known to be important compounds playing a key role in plant-insect interactions (Treutter, 2006). We observed an unclear pattern of expression of some members of this pathway. *Flavanone isomerase* was repressed while *chalcone synthase* was induced. Taken together all these data related to secondary metabolism let us to conclude that *Phoenix canariensis* did not trigger appropriate defenses to promptly counterbalance RPW attacks.

This is might be due to two factors: (1) the RPW-induced genes involved in phenylpropanoids are induced only at late stage when plants are already compromised, (2) it is possible that the genes playing a major roles in the transcriptional regulation of secondary metabolism were not sufficiently enhanced at middle stage of infection (S1).

Other key genes involved in volatile biosynthesis were differentially regulated. The emission of plant volatiles may represent a direct defensive benefit by precluding oviposition (De Moraes et al., 2001) or attracting predators (Kessler and Baldwin, 2001). The important transcript changes that were observed in volatile pathways confirmed that infestations may lead to drastic changes in the volatile profiles detectable by biological methods (Nakash et al., 2000). Novel methods based on detection of induced volatile organic compounds have been recently proposed and should be applied to detect RPW attacks (Dandekar et al., 2010; Martinelli et al., 2015).

**Figure 7:**
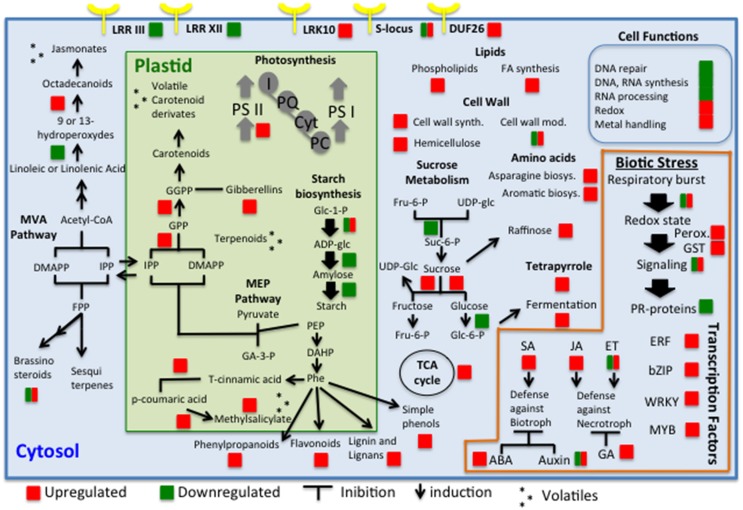
**Global view of the transcriptomic changes in palm leaves in response to RPW attacks**. The genes, pathways, and cell functions that were differentially expressed are indicated with a square (red for upregulated and green for downregulated).

Figure 7 summarized a global view of the main changes that were detected in leaf metabolism considering both analyzed symptom stages. We identified some RPW-induced genes encoding receptor kinases belonging to receptor kinase VIII, DUF26 protein kinases, RLK10-like, and S-locus glycoprotein-like categories. LRR receptor like serine/threonine-protein kinase, S-locus receptor kinase, and TIR-NBS-LRR resistance proteins have been previously found to be upregulated by aphid attacks (Coppola et al., 2013). Interestingly *LRR III* and *LRR XII* were mostly repressed. It is possible that the repression of some of these classes might be involved in a delay or complete lack of signal perception of RPW attacks and consequently in inadequate immune responses to RPW infestations. However, the size and complexity of these gene families make it extremely difficult to understand which family could be involved in susceptible reactions to RPW.

Plant-induced defense responses are modulated by a network of interconnecting hormone crosstalk in which salicylic acid (SA), jasmonic acid (JA), and ethylene (ET) play pivotal roles (Glazebrook, 2001). Jasmonic-related genes were induced by RPW attacks at S2. The synthesis of JA and its precursors and derivatives (collectively termed jasmonates) occurs when plants are wounded. Jasmonates play a central role in regulating defense responses to herbivores (Heil and Ton, 2008). It is expected that a trauma caused by chewing insects or mechanical damage results in the rapid accumulation of JA at the site of wounding. Previous data have demonstrated that the repression of JA responses induces susceptibility to herbivorous insects (Kessler et al., 2004). Here, we observed the upregulation of two key genes (*AOS* and *OPR2*) that are involved in JA biosynthesis during S2. Interestingly, jasmonic-related genes were not upregulated during the middle stage (S1), although their roles in herbivore attack have been demonstrated. This evidence is another clue that demonstrates the inefficient response of *Phoenix canariensis* to RPW infestations. Although crosstalk between defense signaling pathways presents a powerful regulatory instrument, it may also represent a weakness. Here, we observed that a *UDP-glucosyltransferase* that is involved in the conversion of SA to methyl-salicylate was induced during S2. QRT-PCR showed that this gene was significantly upregulated at both stages. It is well known that systemic acquired resistance-against biotroph attacks is antagonist to JA-mediated responses to necrotrophs such as insects. It is possible that the induction of *salicylic acid methyl transferase* would benefit the insect by regulating negatively jasmonic acid-mediated responses. However, previous transcriptomic studies highlighted the upregulation of SA-related genes in response to aphid attacks while the effect on JA-related genes showed a more complex regulation (Coppola et al., 2013). These differences in hormonal plant responses may be due to their diverse type of insect feeding. ABA is connected to the SA-JA-ET network, stimulating JA biosynthesis and antagonizing the onset of SA-dependent defenses. Here, we reported that RPW induced genes that are involved in ABA metabolism and response (*HVA22J, C3HC4-type ring finger*, two *GRAM-domain-containing proteins*). The antagonistic effect of auxin on SA signaling has been documented (Pieterse et al., 2009).

PR proteins were associated with the induction of both locally and systemically induced resistance to pathogens (Van Loon, 1997). Here, we showed that PR genes were differentially regulated by RPW infestations. The majority of PR genes were downregulated implying again a probably weak and inefficacious response to RPW invasion. WRKY proteins are a well-known family of transcription factors involved in biotic stress responses and comprise more than 70 members in *Arabidopsis* (Rushton et al., 2010). Induction of WRKY members have been observed in longitudinal studies of Macrosyphum euphorbiae aphid attacks (Coppola et al., 2013). Upregulation of some members of this family was observed mainly at S2. However, *WRKY40* and *WRKY51* were enhanced also at S1 and might be considered candidate host biomarkers for RPW infestations. *WRKY40* gene has been linked to response to the aphid B. brassicae attacks (Kusnierczyk et al., 2008). A total of 8 WRKY members were significantly enhanced by RPW at stage S2. This important evidence indicates that palms activated a transcription induction of defense responses to RPW larvae feeding although these responses are too late. *WRKY51* transcript was the most abundant (log FD = 7.7). Gao et al. (2011) showed that *WRKY51* plays a key role in stimulating SA-mediated responses and inhibiting JA-inducible defenses, resulting in enhanced resistance to biotrophs but increased susceptibility to necrotrophs. Basing on these data, we may speculate that the induction of this gene as observed in RPW-attacked trees may have negative effects on host plant.

Laccases are enzymes leading to the polymerization of monolignol precursors of lignin. The activation of these genes at middle stage of infestation confirmed that attacked palms responded to RPW invasion. A large number of *peroxidase* genes were induced at stage S2. Two of them were differentially regulated also at stage S1. These transcript changes were expected since peroxidases are well known to be involved in ROS-detoxifying reactions, in the modulation of the redox and Ca^2+^ homeostasis as well as the regulation of defense-related genes (Kawano, 2003). Peroxidases act against biotic attacks in a passive way building up stronger walls or actively producing ROS against pathogens (Moura et al., 2010). When the attacking organism overcomes lignin barriers, peroxidases may be important to isolate the intruder. The evidence that these genes were induced at S1 is a clue that palm are activating defense responses against pathogen colonization.

The ability to rapidly identify RPW infestations is of particular interest because it would allow to rapidly activate the management practices against RPW. In addition, the qRT-PCR analysis of RPW-regulated genes might help in speeding up detection of infestations. Genes upregulated at S1, involved in biotic stress responses, may be further analyzed to check their specificity for RPW attacks such as WRKY40 and WRKY51. A panel of genes may be analyzed in response to abiotic stresses and other typical biotic attacks to check their level of reliability in improving RPW management.

Some possible short-term therapeutic approaches might be proposed. A first approach could focus on boosting JA-mediated defense responses by applying molecules, such as methyl jasmonate, JA, and linolenic acid, which would most likely shift the hormone crosstalk to JA, penalizing SA and allowing trees to localize efforts to pathways that might impair the larvae feeding aptitude. Another possible strategy could be the over-expression of genes playing a key role in the production of compounds that are repellant or not attractive to larvae or adults. Because auxin-responsive genes are antagonists of SA, a worth while experiment would be to test the effects of auxin inhibitor compounds on an attacked tree. It is possibly worthwhile to test the combination of sucrose with agents that stimulate xenobiotic responses through ROS signaling induction as previously suggested (Sulmon et al., 2006). Although an effective RPW management is still absent, the data presented in this work may lead to novel methods of detection of infestations and offer an essential contribution to save palms from RPW attacks, especially in designated World Heritage areas.

## Author contributions

FM and AG conceived and designed the experiments. Experimental work was performed by FM, VF, MA, AG. Data were analyzed by FM, EB, MA, AG, VF, and ML. Manuscript was mainly written by FM in collaboration with AG.

## Funding

The present research work was financially supported by Propalma project funded by the “Ministero delle Politiche Agricole, Alimentari e Forestali.”

### Conflict of interest statement

The authors declare that the research was conducted in the absence of any commercial or financial relationships that could be construed as a potential conflict of interest.
